# First-Principle Studies on Local Lattice Distortions and Thermodynamic Properties in Non-Stoichiometric Thorium Monocarbide

**DOI:** 10.3390/ma16237484

**Published:** 2023-12-02

**Authors:** Qianglin Wei, Lin Zhu, Yiyuan Wu, Yibao Liu, Baotian Wang

**Affiliations:** 1 Engineering Research Center of Nuclear Technology Application, Ministry of Education (East China Institute of Technology), Nanchang 330013, China; qlwei11@ecut.edu.cn (Q.W.); wuyy20@ecut.edu.cn (Y.W.); 2 School of Nuclear Science and Engineering, East China University of Technology, Nanchang 330013, China; 2022120631@ecut.edu.cn; 3 Engineering Technology Research Center of Nuclear Radiation Detection and Application, Nanchang 330013, China; 4 Institute of High Energy Physics, Chinese Academy of Sciences (CAS), Beijing 100049, China; 5 Spallation Neutron Source Science Center, Institute of High Energy Physics, Chinese Academy of Sciences (CAS), Dongguan 523803, China; 6 Collaborative Innovation Center of Extreme Optics, Shanxi University, Taiyuan 030006, China

**Keywords:** non-stoichiometric, first principle, random substitution, lattice distortions, thermodynamic properties

## Abstract

Thorium monocarbide (ThC) is interesting as an alternative fertile material to be used in nuclear breeder systems and thorium molten salt reactors because of its high thermal conductivity, good irradiation performance, and wide homogeneous composition range. Here, the influence of carbon vacancy site and concentration on lattice distortions in non-stoichiometric ThC_1−_*_x_* (*x* = 0, 0.03125, 0.0625, 0.125, 0.1875, 0.25, or 0.3125) is systematically investigated using first-principle calculations by the projector augmented wave (PAW) method. The energy, mechanical parameters, and thermodynamic properties of the ThC_1-*x*_ system are calculated. The results show that vacancy disordering has little influence on the total energy of the system at a constant carbon vacancy concentration using the random substitution method. As the concentration of carbon vacancies increases, significant lattice distortion occurs, leading to poor structural stability in ThC_1−*x*_ systems. The changes in lattice constant and volume indicate that ThC_0.75_ and ThC_0.96875_ represent the boundaries between two-phase and single-phase regions, which is consistent with our experiments. Furthermore, the structural phase of ThC_1−*x*_ (*x* = 0.25–0.3125) transforms from a cubic to a tetragonal structure due to its ‘over-deficient’ composition. In addition, the elastic moduli, Poisson’s ratio, Zener anisotropic factor, and Debye temperature of ThC_1-*x*_ approximately exhibit a linear downward trend as *x* increases. The thermal expansion coefficient of ThC_1−*x*_ (*x* = 0–0.3125) exhibits an obvious ‘size effect’ and follows the same trend at high temperatures, except for *x* = 0.03125. Heat capacity and Helmholtz free energy were also calculated using the Debye model; the results showed the C vacancy defect has the greatest influence on non-stoichiometric ThC_1−x_. Our results can serve as a theoretical basis for studying the radiation damage behavior of ThC and other thorium-based nuclear fuels in reactors.

## 1. Introduction

With increasing demand for electricity and the depletion of uranium resources, the introduction of new nuclear fuels into the fuel cycle has become critical [1,2]. Thorium is a potential convertible nuclear energy resource which is which is approximately three to four times more abundant in the Earth’s crust than uranium [3,4]. In recent years, the development of a thorium fuel cycle has attracted considerable interest worldwide with the purpose of saving uranium reserves and further reducing the production of long-lived minor actinides [5,6]. Actinide carbides are considered to be one of the most promising nuclear fuel materials of generation IV reactors [7,8]. Recently, thorium-based carbides have attracted great attention because of their high melting points, corrosion resistivity, low thermal expansion coefficients, and high thermal conductivity [9]. Therefore, understanding the behavior and properties of thorium-based nuclear fuel is essential for exploring its potential application as nuclear reactor fuel material [10].

The Th-C system has two basic phases: thorium monocarbide (ThC) and thorium dicarbide (ThC_2_) [11]. Cubic (B1-type) ThC has a wide non-stoichiometric region in the carbon sublattice, ThC_1−*x*_ (0 < *x* < 0.33) [12]. Fuel is irradiated in the reactor to produce non-stoichiometric ThC_1−*x*_, which may affect the thermodynamic performance of the fuel [13]. Experimentally, Satow et al. [14] concluded that the lattice parameter increases almost linearly with increasing carbon concentration between the compositions ThC_0.68_ and ThC_0.95,_ while it remains a constant as carbon concentration is lower than ThC_0.68_ and greater than ThC_0.95_. Theoretically, ThC is metallic and structurally stable in the ground state [15]. The formation energy of carbon vacancies in ThC_0.75_ and ThC_0.5_ has shown that ThC can easily create carbon vacancies [16,17]. The relative stabilities of the fan-type and linear structures of gas-phase ThC*_n_* (*n* = 1–7) clusters were also investigated with DFT calculations by Yang et al. [18]. In addition, the high-pressure phase transition of ThC has been studied experimentally and theoretically. Yu et al. [19] experimentally revealed the phase transition of ThC from B1 to P4/nmm at ~58 GPa by synchronous X-ray diffraction. There is no phase transition in ThC under high pressures at 36 GPa [20] and 40–45 GPa [21], but a transitional P4/nmm phase is produced at 60–120 GPa [22], theoretically. 

As an important nuclear energy material, it is well known that defects in ThC are unavoidable due to irradiation damage from high-energy neutrons. Therefore, it is necessary to study the structural stability of non-stoichiometric ThC. Most existing research focuses on stoichiometric ThC and its related phase transition at high pressures. There is less literature available on the lattice distortions and structural stability of non-stoichiometric ThC. In this study, considering the influence of the site and concentration of carbon vacancies on a non-stoichiometric ThC_1−*x*_ (*x* = 0, 0.03125, 0.0625, 0.125, 0.1875, 0.25, or 0.3125) system, its lattice distortions, mechanical parameters, and thermodynamic properties were calculated.

## 2. Calculation Methods and Models

### 2.1. Calculation Method

The calculations were conducted using the VASP package [23] based on DFT [24], employing the projector augmented wave (PAW) method [25]. The exchange-correlation functional used to describe the interactions was the generalized gradient approximation described by Perdew, Burke, and Ernzerh (GGA-PBE) [26]. Twelve electrons (6s^2^6p^6^5f^0^6d^2^7s^2^) for Th and four electrons (2s^2^2p^2^) for C were used as valence electrons in the ThC_1−*x*_ system. Th contains only a small number of 5f states, and it is generally accepted that these states are itinerant: their nature does not need to be corrected with the Hubbard model [27]. Brillouin-zone integrations were carried out with Methfessel–Paxton [28] smearing with a width of 0.2 eV. Through convergence testing, the cutoff energy of atomic wave functions was set to 520 eV for all calculations. The Brillouin zone was sampled with a 9 × 9 × 9 k-point mesh for the 8-atom cell and a 5 × 5 × 5 k-point mesh for the 64-atom supercell using the Monkhorst and Pack (MP) scheme [29]; both meshes were proven to be sufficient for an energy convergence of less than 1.0 × 10^−5^ eV/atom and a force convergence of less than 0.02 eV/Å. The calculation details of p k-point mesh are shown in Appendix A.

### 2.2. Calculation Models

**Figure 1:**
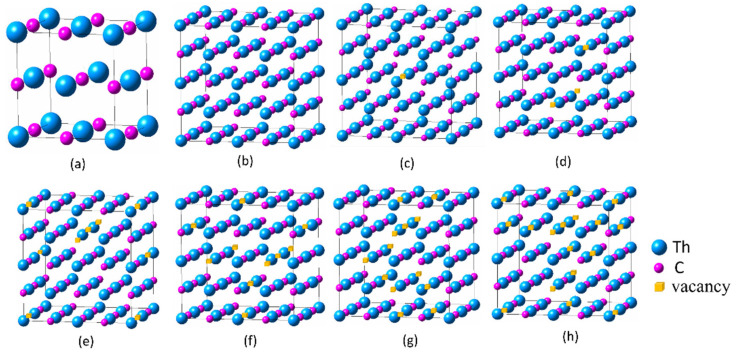
Random substitution models of the 8-atom unit cell structure and 2 × 2 × 2 64-atom supercell structures with the lowest energy in each group of 10. (**a**) *x* = 0, Th_4_C_4_; (**b**) *x* = 0, Th_32_C_32_; (**c**) *x* = 0.03125, Th_32_C_31_; (**d**) *x* = 0.0625, Th_32_C_30_; (**e**) *x* = 0.125, Th_32_C_28_; (**f**) *x* = 0.1875, Th_32_C_26_; (**g**) *x* = 0.25, Th_32_C_24_; (**h**) *x* = 0.3125, Th_32_C_22_.

Under normal temperature and pressure conditions, ThC has the face-centered cubic structure of NaCl (B1), belonging to the $Fm\bar{3}m$ crystal system. The atomic coordinates of Th are (0, 0, 0) and those of C are (0.5, 0.5, 0.5). Lattice parameters (*a*_0_ = 5.3510 Å, α = β = γ = 90°) were obtained from the optimized lattice structure. This is consistent with most theoretical values (5.335–5.388 Å) [7,13,17,22,30,31] and is close to the experimental values of 5.344 Å [14] and 5.430 Å [19]. ThC_1−*x*_ (*x* = 0.03125, 0.0625, 0.125, 0.1875, 0.25, or 0.3125) with specific vacancy concentrations was created by random substitution method obeying Lowenstein’s rule [32], corresponding to the replacement of 1, 2, 4, 6, 8, or 10 carbon atoms with vacancies in a 64-atom supercell, respectively. The 8-atom unit cell structure and typical representatives of the 2 × 2 × 2 supercell structures of ThC_1−*x*_ (*x* = 0–0.3125) are shown in Figure 1.

### 2.3. Crystal and Vacancy Formation Energies

ThC_1−*x*_ can form from metal Th and the most stable graphite $C^{g}$ through the $Th+\left(1-x\right)\;C^{g}\;\to ThC_{1-x}$ reaction. The formation energy per atom in the Th*_k_*C*_l_* supercell, *E_form_*(*ThC*_1−*x*_), is expressed by Equation (1) [33]:

(1) $$E_{form}\left(ThC_{1-x}\right)=\left[E_{tot}\left(Th_{k}C_{l}\right)-kE_{tot}\left(Th\right)-lE_{tot}\left(C^{g}\right)\right]/\left[k+l\right]$$

where *E_tot_*(*Th_k_C_l_*) is the total energy of the Th*_k_*C*_l_* supercell and *E_tot_*(*Th, C^g^*) is the energy per *Th* or *C* atom of each chemical species in its reference state. Here, the reference states are the ground state crystalline phases of *Th* and *C,* namely the thorium α phase and the carbon graphite phase. *k* and *l* are the numbers of Th and C atoms, respectively. According to this definition, a negative *E_form_* means that the ThC_1−*x*_ phase is thermodynamically stable, and the lower the formation energy is, the more stable the state is [34,35].

Vacancy formation energy (*E_vf_*) is obtained using Equation (2) [36]:

(2) $$E_{vf}=\left[E_{tot}\left(ThC_{1-x}\right)+\left(1-x\right)E_{tot}\left(C^{g}\right)-E_{tot}\left(ThC\right)\right]$$

In Equation (2), positive values of *E_vf_* mean that the ThC_1−*x*_ system is still stable as a result of the formation of carbon vacancies, i.e., stable non-stoichiometric phases are formed and vice versa but its stability will reduce. Certainly, such predictions are based only on thermodynamics and do not consider the kinetics of reactions.

### 2.4. Elastic Properties

ThC has cubic crystal system; the space group is 225, which has the highest symmetry degree among all crystal systems. Its independent stiffness matrix element number is only 3, that is *c*_11_, *c*_12_, and *c*_44_. In a cubic crystal system, three independent elastic constants satisfy the following relationship to maintain material stability, as prescribed by the Born–Huang criterion [37]:

(3) $$c_{11}-c_{12}>0,\;c_{11}>0,\;c_{44}>0,\;c_{11}+2c_{12}>0$$

Mechanical parameters, such as the bulk modulus (*B*), shear modulus (*G*), and Young’s modulus (*E*), are calculated to assess the influence of *x* on the structural stability of ThC_1−*x*_. These calculations are carried out by the Voigt–Reuss–Hill approximation [38] using the elastic constants (*c_ij_*) of the crystal system, as shown in Equations (4)–(9) [39].

(4) $$B_{V}=B_{R}=(c_{11}+2c_{12})/3,\;G_{V}=(c_{11}-c_{12}+3c_{44})/5$$

(5) $$G_{R}=5(c_{11}-c_{12})c_{44}/[4c_{44}+3(c_{11}-c_{12})]$$

where *B_V_* and *B_R_* are the Voigt and Reuss bulk moduli, and *G_V_* and *G_R_* are the Voigt and Reuss shear moduli, respectively. *B* and *G* are arithmetic means of the Voigt and Reuss elastic moduli, expressed as:

(6) $$B=(B_{V}+B_{G})/2,\;G=(G_{V}+G_{R})/2$$

(7) E=9BG/(G+3B)

From which Poisson’s ratio (ν) is given by:

(8) ν=(3B−2G)/[2(3B+G)]

and the Zener anisotropy factor (*A*) [13] is given by:

(9) $$A=2c_{44}/(c_{11}-c_{12})$$

The calculation method of the above parameters of other symmetrical structures can be referred to in Ref. [39].

### 2.5. Thermodynamic Properties

Thermodynamic properties are calculated on the basis of the Debye model. Debye temperature (*Ɵ_D_*) is an important fundamental parameter closely related to many physical properties, such as specific heat and melting temperature. At low temperatures, *Ɵ_D_* calculated from elastic constants is the same as that determined from specific heat measurements [13]. We calculated *Ɵ_D_* from the elastic constants using average wave velocity, *v_m_*, by the following common relation [13]:

(10) $$\theta_{D}=\frac{h}{k}{\left[\frac{3n}{4\pi }\left(\frac{N_{A}\rho }{M}\right)\right]}^{1/3}$$

where *v_m_* is calculated by:

(11) $$\upsilon_{m}={\left[\frac{1}{3}\left(\frac{2}{\upsilon_{t}{}^{3}}+\frac{1}{\upsilon_{l}{}^{3}}\right)\right]}^{-1/3}$$

and *v_l_* and *v_t_* are based on the elastic constant:

(12) $$\upsilon_{t}=\sqrt{\frac{3B+4G}{3\rho }}\;,\;\;\;\upsilon_{l}=\sqrt{\frac{G}{\rho }}$$

where *h* is the Planck constant, *k* is the Boltzmann constant, N_A_ is Avogadro’s number, ρ is the density of the crystal in g·cm^−3^, M is the molar mass of the crystal in g·mol^−1^, *n* is the number of atoms in a unit cell, and *v_l_* and *v_t_* are the longitudinal and transverse elastic wave velocities m·s^−1^, respectively.

The volumetric thermal expansion coefficient, α*_V_* (*T*), is then obtained from *V* (*T*) using:

(13) $$\alpha_{V}(T)=\frac{1}{V}(\frac{\partial V(T)}{\partial T})$$

where *V* is the equilibrium volume at 0 K.

In addition, heat capacity and Helmholtz free energy are also calculated on the basis of the Debye model. At low temperatures, heat capacity is integrated to obtain [40]:

(14) $$C_{v}=\frac{12\pi^{4}}{5}Nk_{B}{\left(\frac{T}{\Theta_{D}}\right)}^{3}$$

Helmholtz free energy *A(T)* is then obtained [40]:

A(T)=E−TS

*E* is the energy of ThC_1*x*_ system, *S* is entropy, and *T* is absolute temperature, same as above.

## 3. Results and Discussion

### 3.1. Random Substitution

**Figure 2:**
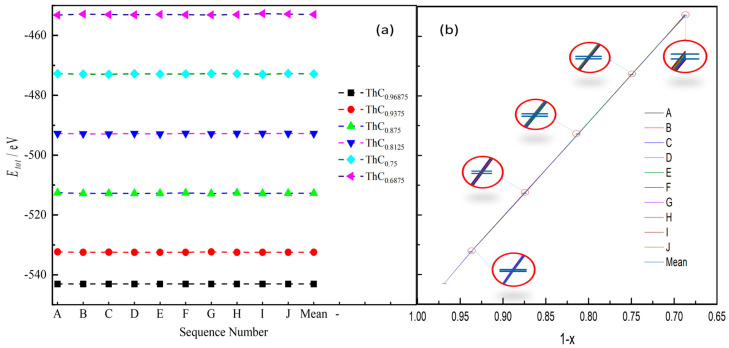
Relationship between carbon vacancy concentration and total energy (E*_tot_*). (**a**) Relationship between ten groups of vacancy configurations (A, B, C, …, I, and J) and *E_tot_* for ThC_1−*x*_ (*x* = 0.03125, 0.0625, 0.125, 0.1875, 0.25, or 0.3125), where ‘Mean’ represents the average value of the group. (**b**) *E_tot_* and standard deviation (amplification in red circle).

In order to find out the effect of carbon vacancy sites on the structural stability of non-stoichiometric ThC_1−*x,*_ ten groups of models (represented by A, B, C, ..., I, and J) of each carbon vacancy concentration in ThC_1−*x*_ (*x* = 0.03125, 0.0625, 0.125, 0.1875, 0.25, or 0.3125) were established using the random substitution method. The total energies of the optimized systems are shown in Figure 2.

As shown in Figure 2a, *E_tot_* of stoichiometric ThC is −552.069 eV, which is lower than the *E_tot_* of all non-stoichiometric ThC_1−*x*_. *E_tot_* of non-stoichiometric ThC_1−*x*_ gradually increases as carbon vacancy concentration increases. These results indicate that ThC is the most stable structure. It is clear from Figure 2b that *E_tot_* is linearly related to carbon vacancy concentration: the smallest standard deviation (SD) reached 0.013% (group A), and the largest SD (group J) does not exceed 1.147%, with a coefficient of variation less than 0.032%. We conclude that vacancy-ordering effects [36] can be ignored in the non-stoichiometric ThC_1−*x*_ system modeled using the random substitution method.

### 3.2. Structural Properties and Formation Energy

The lattice parameters, crystal formation energy, and carbon vacancy formation energy for different carbon vacancy concentrations of ThC_1-*x*_ are shown in Table 1. −Δ*a*/*a_0_* is the change rate of lattice parameter *a*. As seen in Table 1, *a* equals to 5.3512 Å for ThC_0.96875_, 5.3427 Å for ThC_0.875_, and 5.3257 Å for ThC_0.75_, respectively, which are close to the existing experimental data of 5.3470 Å for ThC_0.975_, 5.3429 Å for ThC_0.891_ [41], and 5.31 Å for ThC_0.70_ [11]. When the range of *x* is from 0 to 0.3125, namely in the case of ThC → ThC_0.6875_, −Δ*a*/*a_0_* lies within 0.50%, while the values of α, β, and γ remain relatively stable, at 90.00° ± 0.25°, with distortion rates within ±0.28%. When *x* is less than 0.25, namely in the case of ThC → ThC_0.75_, the ratio of c/*a* is still equal to 1. It indicates that the crystal system can maintain a cubic structure, which is consistent with the result of Shein et al. [36]. When carbon vacancy concentration is increased, in the case of ThC_0.75_ → ThC_0.6875,_ the ratio of c/*a* is equal to 0.997 and 0.994, respectively. These results show that significant structural distortions occur due to the ‘over-deficient’ composition of ThC_1−*x*_. At the same time, the change in lattice volume is calculated; when *x* = 0.125 and 0.3125, then the cell lattice volume decreases by 0.463% and 1.470%, respectively.

**Figure 3:**
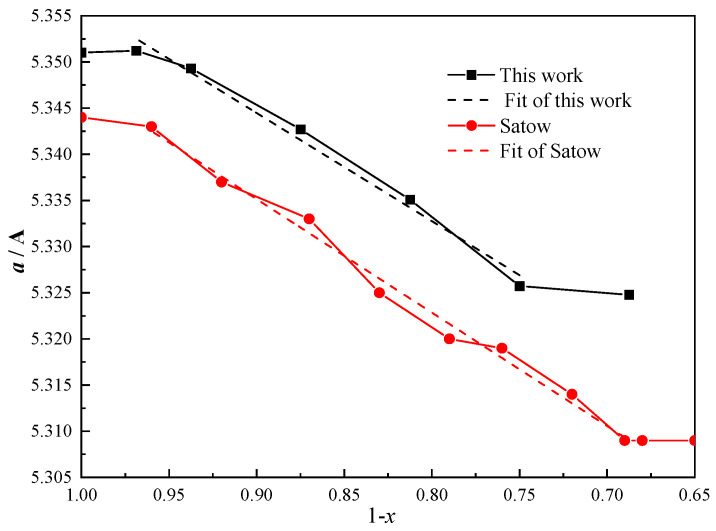
Lattice parameter (*a*) as a function of carbon concentration (1 − *x*) for ThC_1−*x*_ compounds, including a comparison with experimental data.

The relationship between the lattice parameter (*a*) and carbon concentrations (1 − *x*) is shown in Figure 3. The calculated lattice parameters are larger than the experimental values presented by Satow et al. [14], which may have been caused by the GGA algorithm, but they have the same trend. The lattice parameters we calculated decrease almost linearly with increasing carbon concentration between the compositions of ThC_0.75_ and ThC_0.96875_, while constant values were obtained for carbon concentrations lower than ThC_0.75_ and greater than ThC_0.96875_. The values of the boundary range are in good agreement with those of Satow et al. [14], which were obtained by three different experimental methods. This demonstrates the reliability of our calculation results. Furthermore, the occurrence of breaks at ThC_0.75_ and ThC_0.96875_ is considered to indicate the boundaries between the two-phase and single-phase regions. We can infer that ThC_1-*x*_ belongs to the two-phase regions of Th + ThC and ThC + ThC_2_ when the C/Th ratio is lower than 0.75 and greater than 0.96875, respectively. Additionally, this is consistent with the experimental results of non-stoichiometric UC [42].

As shown in Table 1, all *E_form_* of ThC_1−*x*_ containing carbon vacancies are negative, and their values gradually increase as *x* increases. *E_form_* of perfect ThC is −0.444 eV per atom, which was consistent with the calculation results of Shein et al. [12,36]. In addition, perfect ThC is the most stable compound. This result is consistent with the results calculated for uranium monocarbide (UC) [33]. Our calculated *E_form_* of ThC_0.75_ was −0.358 eV per atom, which is also consistent with the result calculated by Shein et al. [36]. Calculation results for other non-stoichiometric ThC_1−*x*_ (x = 0–0.3125) systems have not been reported in the literature.

*E_vf_* is positive, and its value increases as *x* rises, indicating that ThC can easily form carbon vacancies. However, compared with the stoichiometric ThC system, the stability of the non-stoichiometric system is reduced. E*_vf_* of ThC_0.75_ is equal to 0.172 eV, which is very consistent with the results obtained by Daraco et al. [17] using the GGA method with a 64-atom supercell, but lower than those obtained by Wang et al. [7] and Shein et al. [36] using 8-atom supercells, possibly due to the size effect [15].

### 3.3. Elastic Moduli

Elastic moduli are important parameters for characterizing the stability of materials [43]. We calculated the second-order elastic constants (*c_ij_*) at the equilibrium lattice parameter by using the ‘stress-strain’ technique [44], as shown in Table 2. For stoichiometric ThC, *c*_11,_ *c*_12_*,* and *c*_44_ are lower than the result of Aydin et al. [13], while our *c*_11_ and *c*_12_ are in good agreement with the theoretical analysis [15,45]. *c*_44_ is also consistent with [10] and within the range of [13,45].

Table 2 shows that ThC_1−*x*_ (*x* < 0.25) is a cubic crystal. Each elastic constant matrix is determined by three variables (*c*_11_, *c*_12_, and *c*_44_), meeting the material stability condition of the Born–Huang criterion in Equation (3). It indicates ThC_1−*x*_ (*x* = 0–0.25) is structurally stable. When *x* is greater than 0.25, there are six elastic constant variables (*c*_11_, *c*_33_, *c*_44_, *c*_66_, *c*_12_, and *c*_13_), and all of them also satisfy the stability conditions of a tetragonal crystal system: *c*_11_ > 0, *c*_33_ > 0, *c*_44_ > 0, *c*_66_ > 0, (*c*_11_ − *c*_12_) > 0, (*c*_11_ + *c*_33_ − 2*c*_13_) > 0, and [2(*c*_11_ + *c*_12_) + *c*_33_ + 4*c*_13_] > 0 [39]. It is indicated that ThC_1−*x*_ remains stable when *x* is between 0.25 and 0.3125. However, *c*_33_ > *c*_11_ and *c*_66_ > *c*_44_, while *c*_13_ < *c*_12_ for ThC_1−*x*_ (*x* = 0.25–0.3125). Those changes may affect the symmetry of the system. In conclusion, it can be seen that non-stoichiometric ThC_1−*x*_ crystals can still maintain a stable structure despite significant lattice distortion for *x* = 0–0.3125.

**Figure 4:**
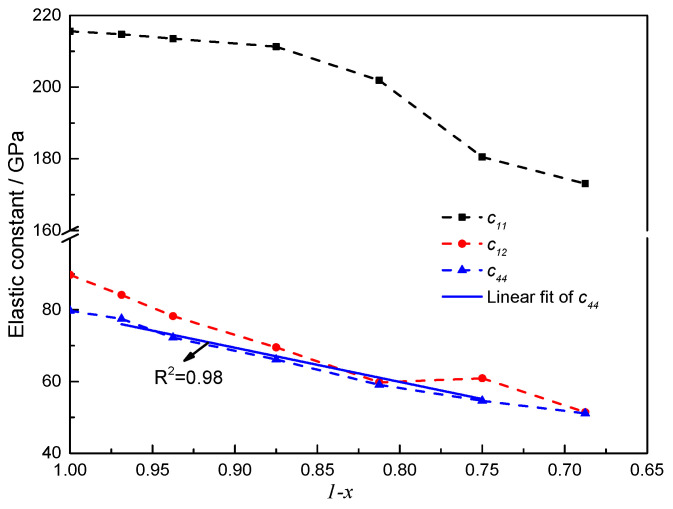
Elastic constants *c*_11_, *c*_12_, and *c*_44_ as a function of (1 − *x*) in ThC_1−*x*_.

All elastic constants decrease as vacancy concentration increases. The values of *c*_11_ are higher than those of *c*_12_ and *c*_44_. *c*_11_ represents elasticity in length, and longitudinal strain produces a change in *c*_11_. *c*_12_ and *c*_44_ are related to elasticity in shape, which is a shear constant, and transverse strain causes a change in shape [13]. As shown in Figure 4, *c*_12_ and *c*_44_ decrease more significantly than c_11_ as carbon vacancy concentration increases, and variation in *c*_44_ is perfectly linear from ThC_0.96875_ to ThC_0.75_. In contrast, shear constant *c*_44_ is important in NaCl structures because it is the modulus most sensitive to next-nearest neighbor, or atom-like, interactions [42]. Thus, *c*_44_ is expected to be the most sensitive to changes in carbon vacancy concentration in ThC_1−*x*_. These results also imply that ThC_1−*x*_, which exists as Th + ThC, can deviate from stoichiometry. This is consistent with the linear variation in lattice parameters with stoichiometry and can be explained by assuming that the structure of hypo-stoichiometric ThC_1−*x*_ primarily contains free thorium, with some vacancies.

**Figure 5:**
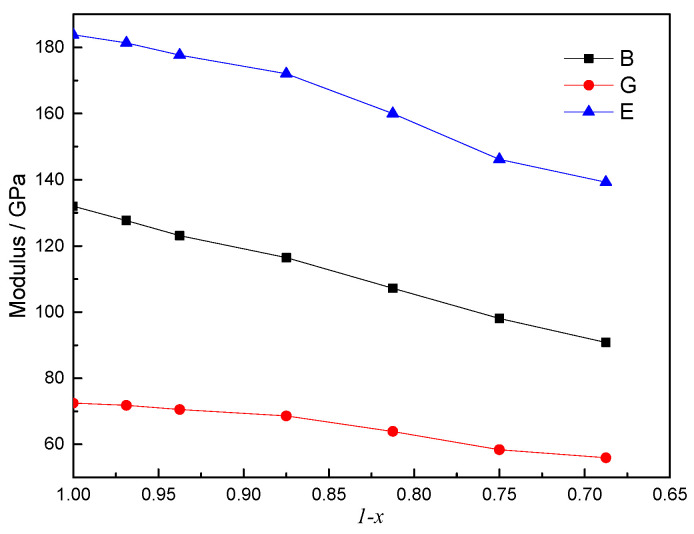
Relationship of volume modulus (B), shear modulus (G), and Young’s modulus (E) with (1 − *x*) in ThC_1−*x*_.

The bulk modulus (*B*), shear modulus (*G*) and Young’s modulus (*E*) were calculated using elastic constants (*c_ij_*) and are shown in Figure 5. For stoichiometric ThC, the calculated bulk modulus is 131.61 GPa, which differs from the experimental value by 11.6 % (147 GPa for ThC_0.95_ at 300 K) [19], but the value agrees quite well (a difference of less than 1.0%) with the data calculated by Aydin et al. [13] (130.2 GPa) and Daraco et al. [45] (131.15 GPa). For non-stoichiometric ThC_0.75_, the calculated bulk modulus is 98.08 GPa, which differs from the experimental data by 10% (109 GPa for ThC_0.76_) [19,20].

As shown in Figure 5, *B*, *G*, and *E* all decrease as *x* increases. *B* changes by 10% for each 10% change in carbon atom concentration from the initial state to the final state. For comparison, Routbort et al. [42] studied the dependence of elastic moduli in UC on stoichiometry and found that the bulk modulus changes by 2% for each 10% change in carbon concentration. This indicates that ThC may be more prone to lattice distortion than UC when carbon vacancy defects are generated.

**Figure 6:**
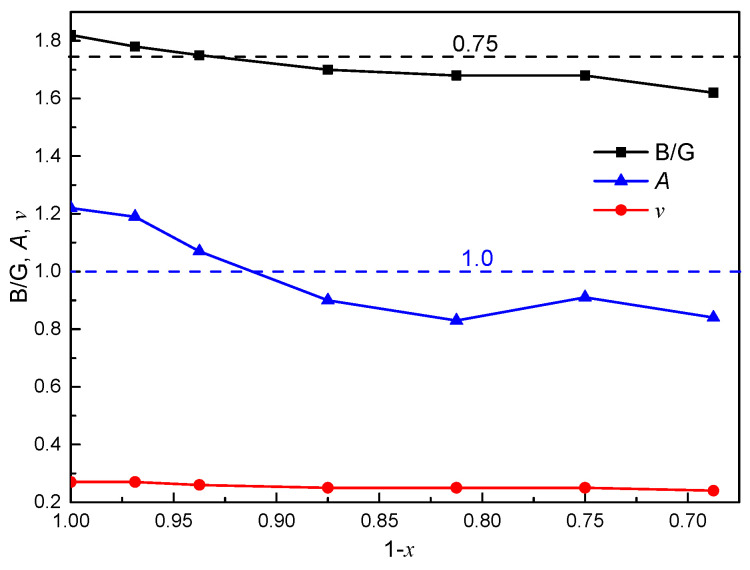
Relationship of the ratio of the bulk modulus to the shear modulus (*B*/*G*), Poisson’s ratio (ν), and Zener anisotropy factor (*A*) with (1 − *x*) in ThC_1−*x*_.

*B*/*G* ratios is also presented in Figure 6. According to the Pugh criterion [46], a material with a *B*/*G* ratio higher than 1.75 is considered ductile, while one with a *B*/*G* ratio lower than 1.75 is considered brittle [47]. We calculated the *B*/*G* ratio of ThC, which was found to be 1.82, thus indicating ductile behavior. The *B*/*G* of non-stoichiometric ThC_1−*x*_ decreases as *x* increases, indicating that the ductility of non-stoichiometric ThC_1−*x*_ decreases with an increase in carbon vacancies. When *x* is larger than 0.9375, non-stoichiometric ThC_1−*x*_ would become brittle because *B*/*G* is less than 1.75.

In addition, Poisson’s ratio (ν) is a very important property for industrial applications because it provides more information about the characteristics of bonding forces rather than elastic constants [48]. As shown in Figure 6, the calculated ν value is equal to 0.27 for ThC at 0 GPa, and agrees with other theoretical values of 0.26 [13] and 0.28 [45]. It is concluded that interatomic forces are dominant in ThC. Moreover, according to the Poisson’s ratio criterion [49], in general, the ν value of a ductile material is approximately 1/3 and is less than 1/3 for a brittle material. As shown in Table 2, all of the values are less than 1/3 and decrease (from 0.27 to 0.24) as *x* increases. These are within the range (from 0.25 to 0.45) for typical metals, except for ThC_0.6875_. This indicates a reduction in ductility due to its ‘over-deficient’ composition.

In contrast, when the Zener anisotropy factor (*A*) is equal to 1.0, it indicates that a material is completely isotropic. There is no evident linear relationship between the Zener anisotropy factor and carbon vacancy concentration. As shown in Table 2, the calculated *A* value of ThC is 1.22, which is greater than the experimental results of 0.97 [45] and 0.98 [13], while the numerical result agrees well with the experimental value of 1.13 [15]. As shown in Figure 6, the values of non-stoichiometric ThC_1−*x*_ (*x* = 0–0.3125) decrease as *x* increases, except for ThC_0.75_, and most of the values are close to 1, indicating that the anisotropy of ThC_1−*x*_ (*x* = 0–0.3125) is small.

### 3.4. Debye Temperature and Thermal Expansion Coefficient

**Figure 7:**
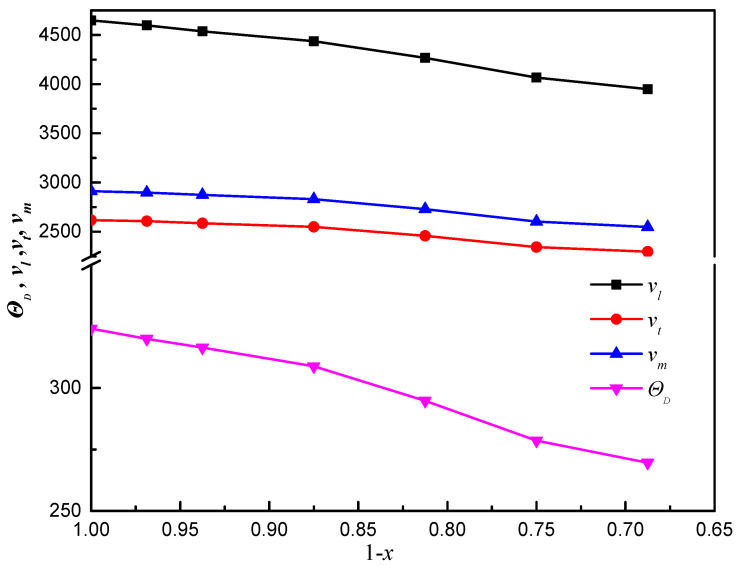
Relationship of the Debye temperature (*Ɵ_D_*, K) and the longitudinal, transverse elastic, and average wave velocities (*ν_l_, ν_t_*, and *ν_m_*, respectively, m·s^−1^) with (1 − *x*) for ThC_1−*x*_.

The relationships of the Debye temperature (*Ɵ*_D_), longitudinal wave velocity (*ν_l_*), transverse elastic wave velocity (*ν_t_*), and average wave velocity (*ν_m_*) with (1 − *x*) for ThC_1−*x*_ are shown in Figure 7. For stoichiometric ThC, the calculated *v_l_*, *v_t_*, and *v_m_* are 2618, 4648, and 2912 m·s^−1^, respectively. The results are in good agreement with those of the elastic constants calculated by Wang et al. (2657, 4709, and 2940 m·s^−1^) [15]. The resulting Debye temperature (*Ɵ_D_*) is 324.1 K, which is in good agreement with that calculated by Wang et al. [15] and Daroca et al. [45] (328 and 311 K, respectively) using the same method of elastic constants. This value is larger than the 298 and 280 K obtained by fitting the isochoric heat capacity curve at low temperatures [45,50] and the experimental value of 262 K [51] obtained on the basis of isobaric heat capacity measurements.

For non-stoichiometric ThC_1−*x*_, *ν_l_*, *ν_t_* and *Ɵ*_D_ decrease as *x* increases. The change in *ν_m_* is almost the same as that in *ν_l_*. *Ɵ*_D_ of non-stoichiometric ThC_1−*x*_ is in the range of 320.0 to 269.6 K when *x* is from 0.03125 to 0.3125.

**Figure 8:**
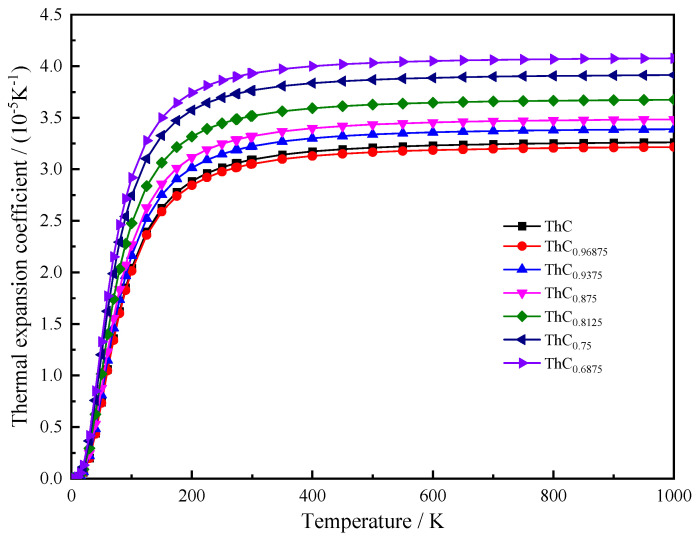
Variation in the thermal expansion coefficient (α) with temperature for 2 × 2 × 2 supercell of ThC_1−*x*_.

The thermal expansion coefficient (α) can be obtained from the temperature derivative of the lattice constant given in Equation (13). Variations in the thermal expansion coefficient with carbon vacancy concentration in the temperature range of 0–1000 K for ThC_1−*x*_ are presented in Figure 8. It is noted that α rapidly increases with T at low temperatures and achieves saturation at approximately 350 K. In addition, α increases as *x* increases at a constant temperature, except for *x* = 0.03125. It is possible that carbon vacancy defects lead to the fracture of the covalent Th-C bond. The lattice volume reduction caused by a single carbon atom defect is not enough to offset the volume swelling caused by the bond fracture. The volume relationship in Table 1 can also explain this phenomenon: although the lattice constants are almost equal, the volume of ThC_0.96875_ is slightly larger than that of ThC. Accordingly, we can see that the α of ThC_0.96875_ is slightly less than that of ThC. As *x* increases further, it leads to increased lattice defects and non-uniformity, resulting in a further increase in the coefficient of thermal expansion.

As for non-stoichiometric ThC_1−*x*_, Ref. [52] reported that the value of the average linear thermal expansion coefficient for ThC_0.96_ was 8.5 × 10^−6^ K^−1^ (using α_V_ = 3α_l_, we obtain α_V_ = 2.55 × 10^−5^ K^−1^) between 974 K and 1174 K. Our average value of α_V_ for the same range of temperature is 3.21 × 10^−5^ K^−1^ for ThC_0.96875_ (for a 64-atom supercell containing four carbon vacancies), and that for ThC_0.76_ was 6.6 × 10^−6^ K^−1^ (α_V_ = 1.98 × 10^−5^ K^−1^) at 974–1104 K. Our average value of α_V_ for the same temperature range is 3.91 × 10^−5^ K^−1^ for ThC_0.75_ (64-atom supercell containing four carbon vacancies). The theoretical values we calculated with the supercells were also larger than those obtained in the experiments. Here, asymmetry plays a more vital role than vibrational amplitude because the symmetry of a crystal also affects its thermal expansion. The worse the symmetry of the crystal, or the more defects in the crystal, the greater the coefficient of thermal expansion. For non-stoichiometric ThC_1−*x*_, the high concentration of carbon vacancies destroys its symmetry, which might be the cause for the overestimation of the thermal expansion coefficient in ThC_1−*x*_.

### 3.5. Heat Capacity and Helmholtz Free Energy

**Figure 9:**
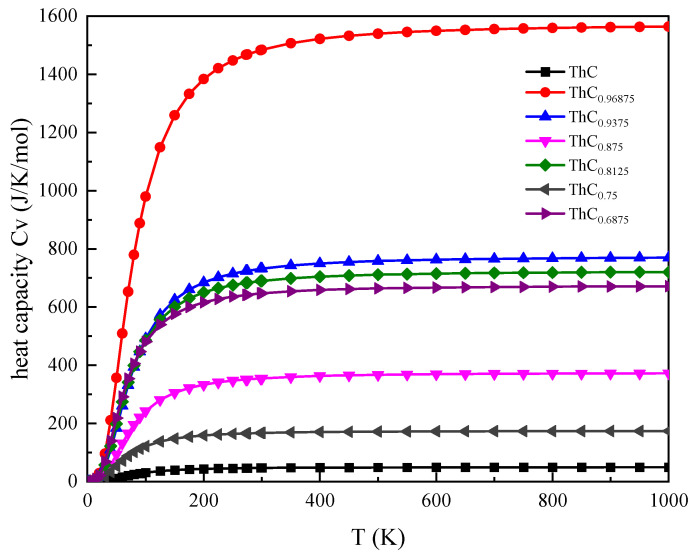
Variation in heat capacity (C_v_) with temperature for 2 × 2 × 2 supercell of ThC_1−*x*_.

**Figure 10:**
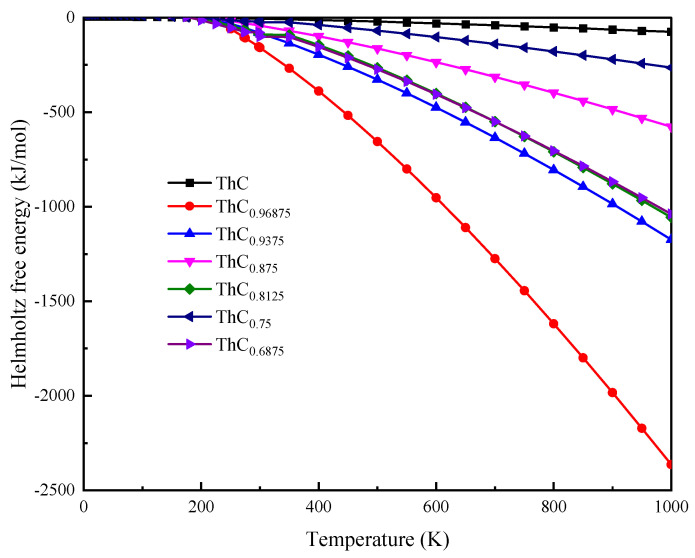
Variation in Helmholtz free energy A(T) with temperature for 2 × 2 × 2 supercell of ThC_1−*x*_.

We also calculated the heat capacity Cv (Figure 9) and Helmholtz free energy A(T) (Figure 10) of ThC_1−*x*_ using the Debye model. Compared with perfect ThC, the C vacancy defect had the greatest influence on the heat capacity and Helmholtz free energy of ThC_0.96875_. This can perhaps be explained with the volume relationship presented in Table 1: although the lattice constants are almost equal, the volume of ThC_0.96875_ is slightly larger than that of ThC. The effect of vacancy defects of other concentrations on heat capacity and Helmholtz free energy is irregular, which is not only related to the relative position of the C vacancy, but also affected by concentration. Although a numerical comparison cannot be made directly due to the inconsistency in the unit of the cell, the changes in the calculated results of free energy are consistent with those of ThC in [8].

## 4. Conclusions

In summary, the crystal energy, elastic parameters, and thermodynamic properties of ThC_1−*x*_ (*x* = 0, 0.03125, 0.0625, 0.125, 0.1875, 0.25, or 0.3125) were studied using density functional theory (DFT) in conjunction with the random substitution method. The results showed that a vacancy-disordering effect is not evident, and it is feasible to adopt the random substitution method. We observed that ‘over-deficient’ carbon vacancies could affect the structural stability of ThC, even though the calculated elastic constants still satisfy traditional mechanical stability conditions. With an increase in carbon vacancy concentration, the lattice constant decreases and is distorted. When *x* is greater than 0.25, ThC_1−*x*_ transforms from a cubic to a tetragonal structure owing to its ‘over-deficient’ composition. Moreover, ‘over-deficient’ carbon vacancies lead to a decline in the toughness and ductility, even leading to brittleness, of non-stoichiometric ThC_1−*x*_. Our calculated results can be used to analyze the stability of ThC fuel in the process of reactor combustion, and the method described in this paper can also be used for theoretical analysis of other thorium-based nuclear fuel in the future.

## Figures and Tables

**Table 1:** Lattice parameters (*a*, c), lattice parameter variation rate (Δ*a/a_0_*), lattice volume (*V*), crystal formation energy (*E_form_*), and carbon vacancy formation energy (*E_vf_*) for ThC_1−*x*_.

Phase	ThC	ThC_0.96875_	ThC_0.9375_	ThC_0.875_	ThC_0.8125_	ThC_0.75_	ThC_0.6875_
*a*/Å	5.3510 5.352 [7] 5.341 [13] 5.344 [14] 5.351 [19] 5.388 [31] 5.3878 [36]	5.3512 5.3470 ^a^ [41]	5.3493	5.3427 5.3429 ^b^ [41]	5.3351 5.325 ^c^ [14]	5.3257 5.31 ^d^ [11] 5.312 [36]	5.3248 5.292 ^e^ [14]
*c/a*	1	1	1	1	1	0.997	0.994
Δ*a/a_0_/%*	0	0	−0.036	−0.159	−0.302	−0.477	−0.494
*V*, unit cell, Å^3^	153.216	153.235	153.071	152.506	151.851	151.052	150.963
*E_form_/*eV	−0.444 −0.55 [12] −0.570 [36]	−0.438	−0.425	−0.407	−0.383	−0.358	−0.337
*E_vf_*/eV	0.000 0.000 [36]	0.013	0.038	0.075	0.123	0.172 0.15 [17] 0.29 ^f^ [7] 0.32 ^f^ [36]	0.215

Given in Refs. [11,14,19,41] are available experimental data. ^a^ for ThC_0.975_ [41]. ^b^ for ThC_0.891_ [41]. ^c^ for ThC_0.80_ [14]. ^d^ for ThC_0.70_ [11]. ^e^ for ThC_0.68_ [14]. ^f^ for the eight-atom supercell.

**Table 2:** Calculated elastic constants ^a^ (*c_ij_*), bulk modulus (B), shear modulus (*G*), B/*G* ratio, Young’s modulus (*E*), Poisson’s ratio (ν), and Zener anisotropy factor (*A*) of ThC_1−*x*_ with other theoretical and experimental data.

Phase	*c*_11_ /GPa	*c*_33_ /GPa	*c*_44_ /GPa	*c*_66_ /GPa	*c*_12_ /GPa	*c*_13_ /GPa	B /GPa	G /GPa	*B/G*	E /GPa	ν	*A*
ThC	215.59 ± 0.60		79.72 ± 0.60		89.74 ± 0.43		131.61	72.49	1.82	183.84	0.27	1.22
ThC [13]	276.4		87.2		99.1		158.2	87.8	1.80	222.2	0.27	0.98
ThC [15]	222.49		80.41		92.03		135.52	74.34	1.82	188.54	0.27	1.13
ThC [45]	222.10		66.12		85.67		131.15	67.10	1.95	171.97	0.28	0.97
ThC_0.96875_	214.73 ± 0.82		76.47 ± 0.58		84.15 ± 0.58		127.68	71.79	1.78	181.38	0.27	1.19
ThC_0.9375_	213.53 ± 0.59		72.23 ± 0.41		78.24 ± 0.41		123.12	70.54	1.75	177.69	0.26	1.07
ThC_0.875_	211.28 ± 0.83		66.14 ± 0.83		68.53 ± 0.59		116.47	68.63	1.70	172.08	0.25	0.90
ThC_0.8125_	201.90 ± 0.78		59.08 ± 0.78		59.75 ± 0.55		107.20	63.93	1.68	159.99	0.25	0.83
ThC_0.75_	180.51 ± 0.72	192.14 ± 0.72	54.62 ± 0.72	55.83 ± 0.72	60.90 ± 0.72	51.99 ± 0.72	98.08	58.40	1.68	146.18	0.25	0.91
ThC_0.6875_	173.06 ± 0.63	180.81 ± 0.63	51.46 ± 0.45	51.95 ± 0.63	51.46 ± 0.45	46.95 ± 0.45	90.87	55.95	1.62	139.26	0.24	0.84

^a^ The errors are from the least-squares fit and only give numerical uncertainty.

**Figure A1:**
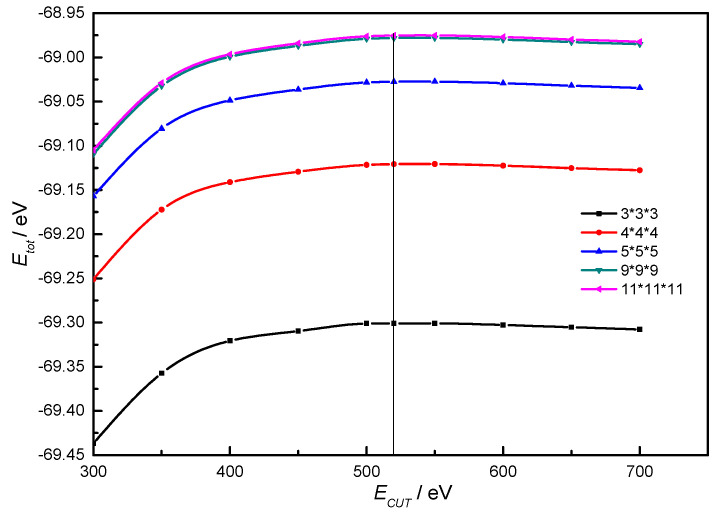
Convergence of total energy (*E_tot_*) as a function of plane wave cutoff energy (*E_cut_*) and k-points.

## Data Availability

The data presented in this study are available on request from the corresponding authors. The data are not publicly available due to ongoing research in the project.

## Appendix A

The convergence of the total energy (*E_tot_*) of the system is determined by two key computational parameters, namely the plane wave cutoff energy (*E_cut_*) and the mesh of k-points, as computed with the MedeA-VASP package. To determine the proper value of *E_cut_* and the number of k-points, *E_tot_* of the ThC system was tested with different *E_cut_* (from 300 to 700 eV with a step of 50 eV) and k-points (3 × 3 × 3, 4 × 4 × 4, 5 × 5 × 5, 9 × 9 × 9, and 11 × 11 × 11). The results are shown in Figure A1. For all k-points, *E_tot_* can be stabilized when *E_cut_* reaches 520 eV. Therefore, *E_cut_* was set as 520 eV. For the same *E_cut_*, *E_tot_* increases as the k-points increase. The difference in *E_tot_* using 9 × 9 × 9 and 11 × 11 × 11 k-point meshes is at most 0.005 eV. Therefore, the Brillouin zone is sampled by a 9 × 9 × 9 Monkhorst–Pack (MP) k-point mesh for 8-atom unit cells in the first step of structural optimization. Then, a 5 × 5 × 5 k-point mesh is selected to calculate the system energy and mechanical properties for 64-atom supercells.
